# HPV-associated cancers among people living with HIV: nationwide population-based retrospective cohort study 2004–21 in Estonia

**DOI:** 10.1093/eurpub/ckae152

**Published:** 2024-10-08

**Authors:** Anna Tisler, Karolin Toompere, Marc Bardou, Jose Diaz, Madleen Orumaa, Anneli Uusküla

**Affiliations:** Institute of Family Medicine and Public Health, University of Tartu, Tartu, Estonia; Institute of Family Medicine and Public Health, University of Tartu, Tartu, Estonia; CIC-P INSERM 1432, Centre Hospitalier Universitaire de Dijon, Dijon, France; Department of Medicine, SUNY Downstate Health Sciences University, Brooklyn, NY, United States; Department of Research, Cancer Registry of Norway, Norwegian Institute of Public Health, Oslo, Norway; Institute of Family Medicine and Public Health, University of Tartu, Tartu, Estonia

## Abstract

Cancers represent the primary cause of mortality among people living with HIV (PLWH). However, comprehensive nationwide data regarding cancer incidence remains limited. Our objective was to evaluate the incidence rates of cancers, particularly those associated with human papillomavirus (HPV), within a nationwide study cohort. Using data from the Estonian Health Insurance Fund and the National Cancer Registry from 2004 to 2021, we calculated standardized incidence ratios (SIRs) for various cancer types among PLWH to compare to the general population with special emphases on HPV-associated cancers. A total of 7011 individuals (65.7% men) diagnosed with HIV were identified. HPV-associated cancers accounted for 21.4% of all incident cancer cases among PLWH. SIRs for HPV-associated cancers were 3.7 [95% confidence interval (CI) 2.2–6.2] among men living with HIV (MLWH) and 5.7 (95% CI 4.0–7.9) among women living with HIV (WLWH). In MLWH, the highest SIRs were for penile 12.5 (95% CI 4.0–38.7), followed by oropharyngeal 3.6 (95% CI 1.7–7.6) and anal–rectal cancers 2.7 (95% CI 1.1–6.4) in comparison to the general population. In WLWH, an increased incidence of cervical (SIR = 5.8, 95% CI 3.9–8.5), oropharyngeal (SIR = 6.1, 95% CI 1.5–24.3), and anal–rectal (SIR = 3.6, 95% CI 1.2–11.2) cancers was observed. A significantly increased risk of AIDS-defining and non-AIDS-defining cancers is reported. We demonstrate a substantially heightened risk of HPV-associated cancers among PLWH compared to the general population, underscoring the imperative for intensified screening and scaled-up vaccination along with improvement in adherence to antiretroviral therapy.

## Introduction

HIV has evolved into a manageable chronic condition with the widespread adoption of antiretroviral therapy (ART) significantly extending the lifespan and overall health of affected individuals. Shifts in the successful management of HIV have led to changes in mortality and morbidity among people living with HIV (PLWH), including increased rates of, and disproportionate burdens related to cancer. Examining cancer burdens among PLWH requires examining multiple forms of cancer, including those historically referred to as AIDS-defining and non-AIDS-defining cancers. Among AIDS-defining cancers, HIV-induced immune suppression has been shown to increase the risk of developing non-Hodgkin’s lymphoma and Kaposi’s sarcoma, two cancers that can signify advanced stages of HIV [1]. Notably, non-AIDS-defining cancers (e.g. Hodgkin’s lymphoma, lung cancer) have become the leading cause of death among PLWH in developed countries [2]. In addition to HIV-induced immune suppression, risk factors for non-AIDS-defining cancer diagnosis include tobacco use (associated with lung, colorectal, stomach, and oropharyngeal cancer) [3, 4], alcohol consumption (colorectal and liver cancer) [5], and viral infections (liver, cervical, anal, penile cancer) [6].

Human papillomavirus (HPV) is an important precursor to developing both AIDS-defining (e.g. cervical) and non-AIDS-defining cancers (e.g. anal). HIV is strongly associated with an adverse impact on the natural progression of HPV, leading to increased acquisition, persistence of HPV infection, and elevated risk of associated lesions in both women and men [7]. Elevated risk is strongly linked to HIV-induced immunosuppression [8], compounded by a higher prevalence of sexually transmitted co-infections [9] and risk behaviors [10]. Cervical, vaginal, vulvar, anal, penile, and oropharyngeal cancers are well-established HPV-associated malignancies, whereas cervical cancer is the most prevalent and extensively researched. Limited studies on HPV-associated cancer incidence are available from Eastern European nations, particularly those with a significant HIV burden [11].

Persistent infection with high-risk HPV is a well-established causal risk factor for cancer; however, numerous behavioral factors mediate this infection status such as anal intercourse, age at initial sexual activity, the number of sexual partners, non-use of condoms and exposure to tobacco smoking [6]. Moreover, the overlapping transmission routes between HIV and cancer-causing viruses such as HPV increase susceptibility to infections in immunocompromised individuals, with recent evidence suggesting a direct contribution of HIV to malignancy development, potentially through chronic inflammation, thus greatly facilitating carcinogenesis [12]. Cervical cancer prevention has become an achievable goal, leading the World Health Organization (WHO) to set targets for its elimination [13]. However, regional variations persist in vaccination and screening coverage [14], particularly among vulnerable women such as living with HIV (WLWH) who often underutilize preventive measures. Organized screening rates decline with age in this population and are significantly influenced by factors such as insurance status, concurrent drug use, and hepatitis C (HCV) infection [15]. Recent comprehensive reviews confirm the safety of HPV vaccines, eliciting a robust initial immune response [16]. While a three-dose schedule is currently recommended for PLWH [17], future research should focus on providing robust evidence for vaccine efficacy after HIV acquisition, its effect on disease endpoints, and the possibility of dosage reduction.

Within Europe, Eastern Europe exhibits the highest HIV rates. Estonia ranks second with an incidence of 18.8 cases per 100 000 individuals, compared to 5.1 cases per 100 000 in the EU/EEA [18]. Limited research has addressed the increased cancer risk in PLWH in Eastern Europe. Nationwide data on HPV-associated cancers in this population is essential for refining preventive and therapeutic strategies [19].

Our study combined data from the population-based nationwide observational cohort of WLWH and MLWH in Estonia to examine the incidence and estimated risk of cancer (HPV, AIDS-defining, non-AIDS-defining cancers) from 2004 to 2021 among PLWH compared with the general population.

## Methods

### Study design and data sources

This retrospective cohort study was based on nationwide population-based electronic health data from the Estonian Health Insurance Fund (EHIF), including health insurance claims, and the Estonian Cancer Registry (ECR). The EHIF is a mandatory health insurance system that covers all citizens in Estonia since 2001 and covers >94% of the Estonian population [20]. ECR includes nationwide cancer cases since 1968 and has shown high completeness and validity [21]. ICD-O-3 coding is used for all cases registered in the ECR which was converted into ICD-10 for the analysis. To define cancer cases among PLWH, we used the unique 11-digit personal identification codes (PIC) assigned to all individuals in Estonia at birth or at immigration to link the data from EHIF with ECR.

### Study population

Data on sex, age, date of HIV diagnosis and of death were obtained from EHIF. HIV status was identified through ICD-10 codes B20-B24, Z21, F02.4, O98.7, R75. Age at the time of the first HIV-related claim, as indicated by the ICD-10 code, was determined for all study participants. Additionally, the participants’ sex was identified by analyzing the initial digit of the PIC, which is specific to males and females. The baseline HIV stage was categorized based on ICD-10 codes, distinguishing between acute infection (B23.0), clinical latency (B23.1, Z21), AIDS diagnosis (F02.4, B20–B24), and cases where the HIV stage was unknown. AIDS diagnosis during the follow-up period was defined by the occurrence of healthcare claims with ICD-10 codes F02.4 or B20–B24. Retention in HIV care was assessed by stipulating a minimum of two HIV-related physician visits within a continuous 12-month period throughout the study duration [22]. Furthermore, drug use disorders were identified through ICD-10 diagnoses, encompassing codes F10–F19, T40, and Y12. Coinfection with HCV (B17.1 or B18.2) and sexually transmitted infections (STI) (A50–64) are reported. A cervical cancer screening episode was defined based on a health claim with the ICD-10 diagnosis code Z12.4 and service code of HPV or cytology test. All individuals diagnosed with HIV in the years 2004–21 were included and were followed until the end of the study (31 December 2021), outcome or death, whichever came first.

### Cancer data

The primary study outcome included the crude cancer incidence rate per 100 000 for selected cancers. Additionally, the standardized incidence ratio (SIR) was assessed to depict the ratio of cancer incidence in the PLWH compared to the incidence in the general population. Incident cancer cases with the date of diagnosis were identified through ECR data. The types of cancer were classified as HPV-associated, AIDS-defining, and non-AIDS-defining cancers. The previously documented definitions of the cancer categories were employed [23, 24].

HPV-associated cancers included cervical (C53), vulva (C51), penile (C60), anal and rectal (C20–21), oropharyngeal (C01.9, 02.4, 02.8, 05.1–05.2, 09.0–09.1, 09.8–09.9, 10.0–10.4, 10.8–10.9, 14.0, 14.2, 14.8). AIDS-defining cancers included Kaposi sarcoma (C46), cervical cancer (C53), and non-Hodgkin lymphoma (C82–C86, C96). Non-AIDS-defining cancers included brain and central nervous system (C70–C72), urinary tract (C64–C68), breast (C50), colorectal (C18–C20), Hodgkin lymphoma (C81), liver, bile duct, and pancreatic (C22–C25), lung and tracheal (C33, C34), lip, oral cavity, and pharyngeal (C00–C14), stomach (C16), testis (C62), thyroid (C73), prostate (C61), larynx (C32). The analysis included one occurrence of each cancer type per individual and those diagnosed after the HIV diagnosis.

### Additional data sources

Mean annual population data by age groups were derived from Statistics Estonia [25]. The overall cancer data for the general population were sourced from the Health Statistics and Health Research Database [20].

The institutional review board of the University of Tartu approved the study protocol 384/M-26.

This study followed the Strengthening the Reporting of Observational Studies in Epidemiology (STROBE) reporting guideline [26].

### Statistical analysis

Descriptive statistics [i.e. proportions, means, standard deviations (SDs)] for length of follow-up time and study cohort characteristics were reported. The crude rate of cancer incidence among individuals diagnosed with HIV across all age groups was presented as the number of cases per 100 000 person-years. The follow-up person-years at risk were estimated from the HIV diagnosis date to the date of cancer diagnosis, date of death, or 31 December 2021, whichever came first.

The cancer incidence in the PLWH cohort, compared with the general population was compared through indirect standardization and assessed by SIR. SIRs were expressed as the ratio of observed to expected number of cases. The expected number of cases was calculated by multiplying the number of person-years at risk stratified by 5-year age groups and 5-year calendar periods by corresponding cancer in the general population. The 95% confidence intervals (CIs) for the SIR were computed assuming that the observed number of cases followed a Poisson distribution. The stratification of the analysis by sex aimed to address variations in susceptibility. STATA software, version 17 was used for statistical analyses.

## Results

The study cohort consisted of 7011 individuals (65.7% males) who were diagnosed with HIV between 2004 and 2021. The mean age for HIV diagnoses among men and women was 32.0 and 28.5 years, respectively. The majority of PLWH (men: 73.6%, women: 76.8%) were in the clinical latency stage at the start of follow-up, and (men: 27.2%, women: 20.2%) were considered retained in HIV care and one-third (men: 28.7%, women: 28.6%) were diagnosed with AIDS during the study period. More MLWH (58.2%) than WLWH (38.7%) had diagnoses codes indicating drug use disorders, concomitant HCV infection (men: 58.9%, women: 44.6%), and STIs (men: 10.8%, women: 38.4%) (Table 1). Throughout the study period, 44% of WLWH aged 21 and older did not receive either cytology or HPV testing. Furthermore, only 11.3% were consistently followed up every five years for cervical cancer screening.

**Table 1.** Characteristics of PLWH in Estonia, 2004–21

Characteristics	MLWH (*N* = 4603)	WLWH (*N* = 2408)
Age at HIV diagnoses, mean (SD)	32.0 (10.5)	28.5 (12.6)
Age at HIV diagnosis, *n* (%)		
0–4	98 (2.1)	96 (3.9)
5–9	15 (0.3)	12 (0.5)
10–14	16 (0.3)	15 (0.6)
15–19	115 (2.5)	276 (11.5)
20–24	700 (15.2)	697 (28.9)
25–29	1275 (27.7)	506 (21.0)
30–34	934 (20.3)	280 (11.6)
35–39	600 (13.0)	169 (7.0)
40–44	347 (7.5)	108 (4.5)
45–49	239 (5.2)	87 (3.6)
50–54	123 (2.7)	57 (2.4)
55–59	67 (1.5)	43 (1.8)
60–64	42 (0.9)	21 (0.9)
65–69	16 (0.3)	19 (0.8)
70–74	13 (0.3)	6 (0.2)
75–79	3 (0.06)	10 (0.4)
80–84	0	5 (0.2)
85+	0	1 (0.04)
Period of HIV diagnosis, *n* (%)		
2004–09	2346 (50.9)	1456 (60.5)
2010–15	1492 (32.4)	650 (26.9)
2016–21	765 (16.6)	302 (12.5)
Follow-up years, mean (SD) HPV-related cancers	8.6 (5.4)	10.6 (5.4)
Follow-up time total, person-years HPV-related cancers	39 501	25 599
HIV stage at baseline, *n* (%)		
Clinical latency	3389 (73.6)	1849 (76.8)
Acute	138 (2.9)	75 (3.1)
AIDS	966 (20.9)	377 (15.7)
Unknown	110 (2.4)	107 (4.4)
AIDS diagnosis during the study period, *n* (%)	1320 (28.7)	688 (28.6)
HIV care retention (yes), *n* (%)	1252 (27.2)	486 (20.2)
HCV (yes), *n* (%)	2710 (58.9)	1075 (44.6)
Drug use disorder (yes), *n* (%)	2678 (58.2)	932 (38.7)
STI (yes), *n* (%)	496 (10.8)	924 (38.4)
WLWH without cytology or HPV test during the whole study period (aged 21+), *n* (%)		955 (44.3)
WLWH covered by cytology and HPV test every 5 years (aged 21+), *n* (%)		244 (11.3)

From 2004 to 2021, a total of 229 cancer cases were diagnosed among individuals living with HIV in Estonia. Of these diagnosed cancers, 185 cases (115 MLWH, 70 WLWH), occurring in 179 individuals were specifically identified as HPV-associated cancers, AIDS-defining, or non-AIDS-defining cancers. HPV-associated cancers accounted for 21.4% (49/229) of all incident cancer cases among PLWH, while AIDS-defining and non-AIDS-defining cancers account for 31.9% (73/229) and 46.7% (107/229) of all cases, respectively.

### HPV-associated cancers

The mean time between HIV diagnoses and HPV-associated cancer diagnoses was 8.3 years for MLWH and 7.3 years for WLWH. The total follow-up years for 49 cases were 39 501 in MLWH and 25 599 in WLWH, with a mean (SD) of 8.6 (5.4) and 10.6 (5.4), respectively. The crude HPV cancer incidence rate was 37.9 (95% CI 22.9–62.9) per 100 000 person-years in MLWH and 132.8 (95% CI 94.9–185.9) per 100 000 person-years in WLWH. The most common HPV-associated cancer among MLWH was an oropharyngeal cancer incidence rate of 17.7 (95% CI 8.5–37.2) followed by anal–rectal with an incidence of 12.7 (95% CI 5.3–30.4) and penile 7.6 (95% CI 2.5–23.5) per 100 000 person-years. Among WLWH incidence rate per 100 000 person-years of cervical cancer was 105.8 (95% CI 72.5–154.2) followed by anal–rectal 11.6 (95% CI 3.8–36.1). Vulvar and oropharyngeal had the same incidence of 7.8 (95% CI 1.9–31.0) (Table 2).

**Table 2.** Rates of incident cancer cases per 100 000 person-years among PLWH in Estonia 2004–21

Cancers	MLWH, *n*	Person time	Incidence rate (95% CI)	WLWH, *n*	Person time	Incidence rate (95% CI)
HPV-associated	15	39 501.0	37.9 (22.9–62.9)	34	25 598.9	132.8 (94.9–185.9)
Anal-rectal	5	39 515.5	12.7 (5.3–30.4)	3	25 753.1	11.6 (3.8–36.1)
Cervical				27	25 536.8	105.8 (72.5–154.2)
Oropharyngeal	7	39 513.9	17.7 (8.5–37.2)	2	25 762.0	7.8 (1.9–31.0)
Penile	3	39 522.9	7.59 (2.45–23.54)			
Vulva				2	25 760.0	7.8 (1.9–31.0)
AIDS-defining	35^a^	39 419.0	88.8 (63.8–123.7)	38^b^	25 584.9	148.5 (108.1–204.1)
Kaposi’s sarcoma	2	39 516.7	5.1 (1.3–20.2)			
Non-Hodgkin’s lymphoma	33	39 427.9	83.7 (59.5–117.7)	11	25 733.7	42.7 (23.7–77.2)
Non-AIDS-defining	77	39 370.1	185.4 (147.4–233.2)	30	25 675.1	112.9 (78.5–162.5)
Hodgkin’s lymphoma	5	39 511.5	12.7 (5.3–30.4)			
Brain and CNS	7	39 507.6	17.7 (8.4–37.2)	2	25 742.3	7.8 (1.9–31.1)
Urinary tract	3	39 499.1	7.6 (2.4–23.5)			
Breast				8	25 741.5	31.1 (15.5–62.1)
Colorectal	8	39 514.3	20.2 (10.1–40.5)	5	25 741.3	19.4 (8.1–46.7)
Liver, bile duct, pancreas	6	39 520.8	15.2 (6.8–33.8)	3	25 762.8	11.6 (3.8–36.1)
Lung	14	39 515.7	35.4 (20.9–59.8)	7	25 758.4	27.2 (12.9–57.0)
Lip, oral cavity, and pharyngeal	8	39 512.6	20.2 (10.1–40.5)	2	25 762.0	7.8 (1.9–31.0)
Stomach	8	39 513.1	20.2 (10.1–40.5)	1	25 753.7	3.9 (0.5–27.6)
Thyroid	2	39 524.9	5.0 (1.3–20.2)	2	25 758.8	7.8 (1.9–31.0)
Prostate	12	39 476.8	30.4 (17.3–53.5)			
Larynx	2	39 517.6	5.1 (1.3–20.2)			
Testis	2	39 520.4	5.1 (1.3–20.2)			
All cancers^c^	115	39 261.2	280.2 (232.4–337.7)	70	25 492.7	270.7 (213.8–342.7)

a Kaposi’s sarcoma and non-Hodgkin’s lymphoma.

b Non-Hodgkin’s lymphoma and cervical cancer.

c Without overlapping.

**Figure 1.**
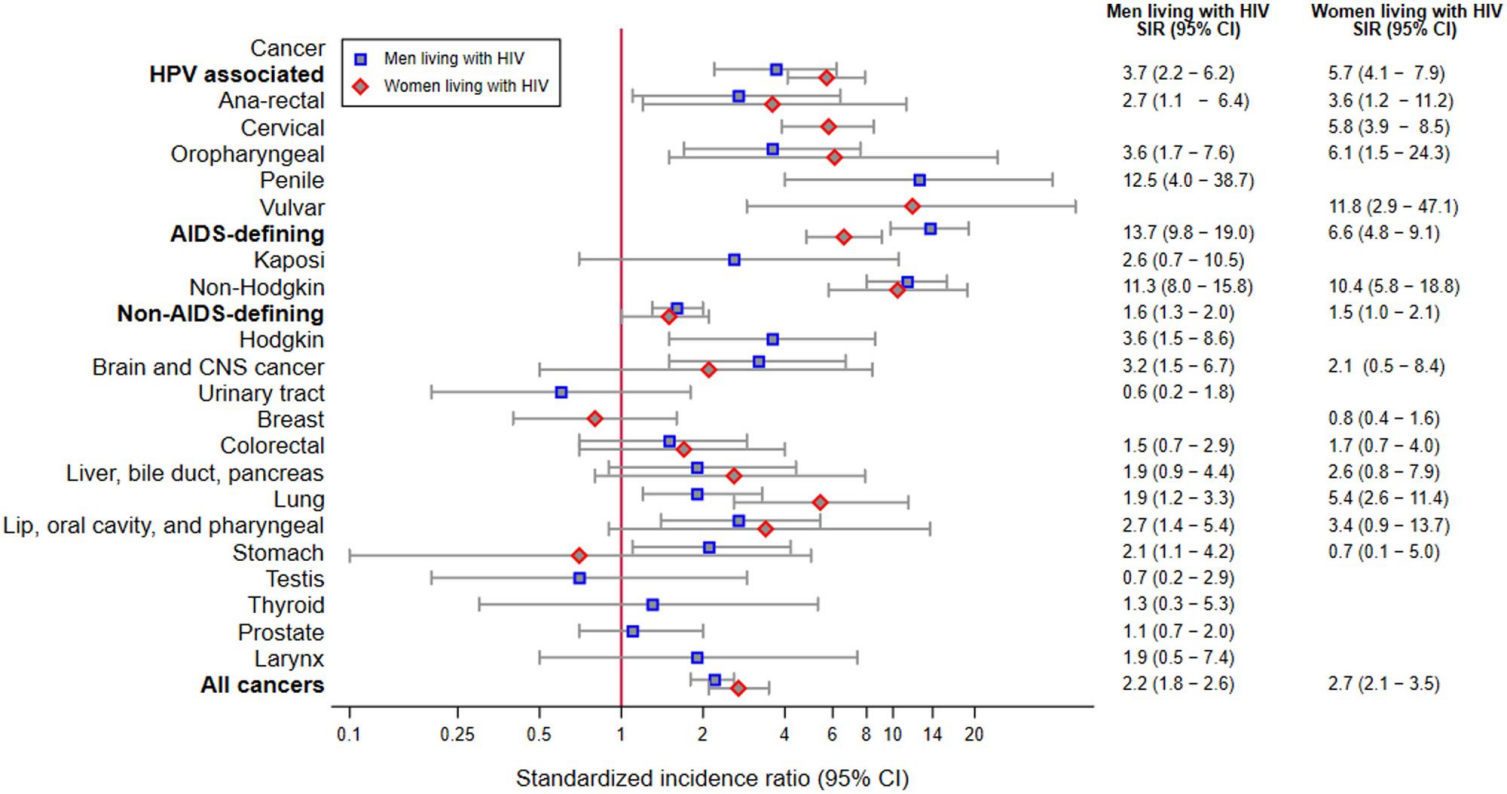
Cancer SIRs comparing PLWH to the general population in Estonia, 2004–21.

The SIRs of total HPV-associated cancer were 3.7 (95% CI 2.2–6.2) and 5.7 (4.0–7.9) among MLWH and WLWH, respectively, indicating a significantly higher cancer incidence than in the general population. In WLWH, there was an increased incidence of oropharyngeal (SIR = 6.1, 95% CI 1.5–24.3), cervical (SIR = 5.8, 95% CI 3.9–8.5), vulvar (SIR = 11.8, 95% CI 2.9–47.1), and anal–rectal (SIR = 3.6, 95% CI 1.2–11.2) cancers compared with the general female population (Fig. 1). Among MLWH, penile (SIR = 12.5, 95% CI 4.0–38.7), oropharyngeal (SIR = 3.6, 95% CI 1.7–7.6), and anal–rectal (SIR = 2.7, 95% CI 1.1–6.4) were each significantly higher in comparison to the general male population.

### AIDS-defining and non-AIDS-defining cancers

There were 77 and 107 cases of AIDS-defining and non-AIDS-defining cancers, respectively (Table 2). The overall SIR indicated a significant increase among PLWH in comparison to the general population for AIDS-defining cancers SIR 13.7 (95% CI 9.8–19.0) and 6.6 (95% CI 4.8–9.1) and for non-AIDS-defining cancers SIR 1.6 (95% CI 1.3–2.0) and 1.5 (95% CI 1.0–2.1) among MLWH and WLWH. For the majority of cancers, we found no statistically significant difference between incidences between PLWH and the general population. This is likely due to a small number of cases (<10) for many cancers among PLWH. Still, SIR point estimates for breast, testis, and urinary tract cancers were lower in PLWH than in the general population.

## Discussion

This study utilized nationwide data from Estonia and documented an increased risk of HPV-associated, AIDS-defining, and non-AIDS-defining cancers among both MLWH and WLWH in comparison to the general population.

Our study found that PLWH have a roughly three-fold increased risk of developing HPV-associated cancers compared to the general population. This elevated risk applies across various HPV-related cancers in both men and women. However, it is important to note that the specific risk differ between cancer sites and genders.

We observed a gradient in risk for HPV-related cancers among PLWH. Penile and vulvar cancers showed the strongest increase in risk, with PLWH experiencing roughly 12 times the risk compared to the general population. WLWH had a 6-fold increased risk of cervical and oropharyngeal cancers compared to their HIV-uninfected counterparts. MLWH had a 3.6 times greater risk of oropharyngeal cancer compared to men in the general population. The risk of anal cancer among PLWH was approximately three times higher than in the general population, with less marked gender difference.

The increased risk of HPV-related cancers was comparable to that reported in previous studies [27–30].

The disparity in risk between cervical and vulvar cancer may potentially stem from the historical emphasis on preventing cervical cancer through screening, coupled with the comparatively lower number of vulvar cancer cases detected. Consequently, potential abnormalities or early signs of cancer in the vulva or vagina may remain undetected for longer periods, heightening the risk of cancer progression.

The parallels in the natural progression of anal and cervical cancer suggested the feasibility of employing comparable preventive strategies for anal cancer. Treating high-grade anal lesions PLWH effectively reduces the risk of progression to anal cancer, this evidence has influenced recent guidelines on anal cancer screening by the International Anal Neoplasia Society, albeit limited capacity for high-resolution anoscopy poses a significant constraint [31]. Additionally, in WLWH the risk is attributed to HPV cross-site transmission, facilitated by genital–anal anatomical factors [29].

Furthermore, although we had few cases of non-AIDS-defining cancers to analyze, we found a substantially increased risk of lip, lung, and stomach cancers among PLWH. The risk factors for oral HPV acquisition in the oropharynx and its incidence among PLWH, as well as HPV dynamics compared to HIV-negative individuals, are not well understood. However, tobacco smoking is a known and documented predictor of oropharyngeal cancer [6]. The proportion of daily smokers in Estonia stands at 18%, surpassing the EU average and peaking in areas with the highest HIV prevalence [32]. Similarly, PLWH report high levels of tobacco use globally, indicating a need to pair tobacco cessation programs and lung cancer screening, including low-dose computer tomography [33].

For AIDS-defining cancers, our findings reveal a notably high risk among both MLWH (SIR = 13.7, 95% CI 9.8–19.0) and WLWH (SIR = 6.6, 95% CI 4.8–9.1) which runs counter to the overarching trend of declining incidence attributed the improved HIV therapy [34]. These findings could potentially be linked to suboptimal HIV treatment coverage rates (MLWH: 27.2%, WLWH: 20.2%) observed in our data.

Although our study is based on Estonian data, its findings hold relevance for cancer prevention and management among PLWH worldwide. Despite progress in ART and vaccination and screening programs, PLWH still confront heightened risks of HPV-associated, AIDS-defining, and non-AIDS-defining cancers. To mitigate these risks, effective strategies addressing lifestyle factors, promoting smoking cessation programs, and implementing comprehensive prevention initiatives beyond ART initiation are crucial. Scaling up HIV treatment access and retention in care is critical in reducing the excess burdens experienced by PLWH. Our study underscores the urgent need for improved cancer screening in Europe, particularly for high-risk groups, due to low coverage rates and limited policies [15, 35]. Targeted interventions like HPV-FASTER concept [36] can enhance cancer elimination and focus efforts on fostering active engagement across the entire screening process to ensure timely identification and treatment for those at higher risk. Implementing a gender-neutral cancer prevention strategy, will decrease population-wide HPV infections, counter misinformation, reduce vaccine-related stigma, and advance gender equity. Current HPV vaccines are prophylactic and do not clear existing infections, underscoring the need for preadolescent vaccination before sexual activity and HIV infection. However, given the high risk of HPV-related diseases in PLWH, vaccination remains beneficial even after sexual debut, as it can help protect against new HPV infections.

Novel treatments, such as immunotherapy and therapeutic vaccines would be needed to improve cancer survival until HPV vaccination reaches population coverage and boosts the global fight against these cancers [37]. Furthermore, community-based intervention design, implementation, and dissemination, in collaboration with PLWH, are essential to address the specific context and needs of this population.

Study limitations warrant consideration. First, other relevant risk factors for HPV cancers, such as smoking, parity, and long-term oral contraceptive use were not available potentially leading to an overestimation of risk. The study relied on claims data, and consequently, information on viral suppression, including CD4 count and HIV viral load, was not accessible. Cancers that were not diagnosed among PLWH (over the period of study) such as vaginal, esophageal, and myeloma are also not included. We did not differentiate between incident and prevalent cancer risks. Nevertheless, considering the mean time from HIV to cancer diagnoses, thus it is plausible to speculate that these cases are predominantly incident in nature. Additionally, we do not have HPV vaccination data specific to HIV status. However, only 0.09% of the Estonian population was vaccinated between 2008 and 2013, and a national HPV vaccination program was launched in 2018. Our focus on Estonia may under-represent differences in cervical cancer between PLWH and the general population. Estonia is a high-income country with population-wide cervical cancer screening that has established accessibility to treatment for women living with HIV. Still, these findings may support expanding screening and treatment access in other countries with greater differences in cervical cancer risk by HIV status. Additionally, we were unable to present estimates stratified by age, as there were insufficient numbers of cases in a majority of age groups. For the same reason, it was not feasible to provide a distribution by cancer stages and time-specific estimates. Still, a key strength of our study lies in its population-based design and utilizes robust nationwide data, encompassing all individuals living with HIV in Estonia.

## Conclusion

Our study presents estimations of cancer risk, particularly highlighting the elevated risk of HPV-associated cancers among PLWH in Estonia. We emphasize a significantly heightened risk compared to the general population, emphasizing the urgent need to enhance prevention efforts, including gender-neutral vaccination, achieving recommended screening rates, and integrating cancer prevention into HIV care. Addressing risky behaviors through targeted interventions is crucial for reducing the overall cancer risk. These efforts are pivotal in tackling significant public health challenges and reducing the current disparities in disease burden.

## Data Availability

There are legal restrictions on sharing a de-identified data. According to legislative regulation and data protection law in Estonia, the authors cannot publicly release the data received from the health data registers in Estonia. The data can be requested by completing the application in order to carry out research or an evaluation of public interest from EHIF https://www.tervisekassa.ee/en and ECR https://en.tai.ee/en/r-and-d/registers/estonian-cancer-registry. Key points People living with HIV (PLWH) experience an increased risk of cancers. We used a nationwide population-based cohort to estimate the risk of cancer among PLWH. Human papillomavirus-associated cancer risk among PLWH was higher in comparison to the general population. Intensified screening and scaled-up vaccination along with improvement in adherence to antiretroviral therapy are crucial.
